# Bioaccumulation of total mercury in the earthworm *Eisenia andrei*

**DOI:** 10.1186/s40064-016-2282-6

**Published:** 2016-05-20

**Authors:** Shirley Le Roux, Priscilla Baker, Andrew Crouch

**Affiliations:** SensorLab, Chemistry Department, University of the Western Cape, PB X17, Bellville, 7535 South Africa; Faculty of Science, University of the Witwatersrand, Private Bag 3, Johannesburg, 2050 South Africa

**Keywords:** Bioavailability, Bioaccumulation, Bioaccumulation factor, Humic acid, Earthworms, Soil, Mercury

## Abstract

Earthworms are a major part of the total biomass of soil fauna and play a vital role in soil maintenance. They process large amounts of plant and soil material and can accumulate many pollutants that may be present in the soil. Earthworms have been explored as bioaccumulators for many heavy metal species such as Pb, Cu and Zn but limited information is available for mercury uptake and bioaccumulation in earthworms and very few report on the factors that influence the kinetics of Hg uptake by earthworms. It is known however that the uptake of Hg is strongly influenced by the presence of organic matter, hence the influence of ligands are a major factor contributing to the kinetics of mercury uptake in biosystems. In this work we have focused on the uptake of mercury by earthworms (*Eisenia andrei*) in the presence of humic acid (HA) under varying physical conditions of pH and temperature, done to assess the role of humic acid in the bioaccumulation of mercury by earthworms from soils. The study was conducted over a 5-day uptake period and all earthworm samples were analysed by direct mercury analysis. Mercury distribution profiles as a function of time, bioaccumulation factors (BAFs), first order rate constants and body burden constants for mercury uptake under selected conditions of temperature, pH as well as via the dermal and gut route were evaluated in one comprehensive approach. The results showed that the uptake of Hg was influenced by pH, temperature and the presence of HA. Uptake of Hg^2+^ was improved at low pH and temperature when the earthworms in soil were in contact with a saturating aqueous phase. The total amount of Hg^2+^ uptake decreased from 75 to 48 % as a function of pH. For earthworms in dry soil, the uptake was strongly influenced by the presence of the ligand. Calculated BAF values ranged from 0.1 to 0.8. Mercury uptake typically followed first order kinetics with rate constants determined as 0.2 to 1 h^−1^.

## Background

Organic matter strongly affects the binding of mercury ions in the environment and affects the mobility and bioavailability of this metal in sediments (Gismera et al. 2007). Mercury (and also other metals) can be reintroduced into the aquatic systems if the solubility, mobility and bioavailability changes as a result of a change in different environmental factors such as pH, salt concentration, the presence of complexing agents, and temperature. Sediments can be a long-term source of Hg to surface waters. Sediments are an important location as storage reservoirs for elemental mercury and facilitate Hg methylation, resulting in high concentrations of the more toxic monomethylmercury (CH_3_Hg^+^) in organisms (Inza et al. 1997; Lawrence and Mason 2001; Burton et al. 2006; Windmöller et al. 2015).

The toxicity of Hg to terrestrial invertebrates and its effects on survival, reproduction and growth is not well documented although such information is important for risk assessment from Hg pollution in terrestrial ecosystems. The toxicity of Hg^2+^ is strongly linked to its affinity for the sulphydryl groups, which are found in most proteins (Gudbrandsen et al. 2007). Earthworms in particular, have the ability to bioaccumulate toxins and can concentrate them internally to high levels. In turn they form the basis of many food chains, thereby passing these high levels on to the wildlife that feed on them. This poses a serious risk of secondary poisoning of these predators due to bio-magnification (Kamitani and Kaneko 2007). Because of the intricate relationship between earthworms and the soil and its contaminants, they can serve as useful biological indicators of soil contamination (Veiga et al. 1999; Dai et al. 2004).

Earthworms eat their way through soil, digest it and deposit it as waste, thereby aerating and mixing the soil (Ernst et al. 2008). This process enhances the uptake of nutrients in the soil by plants. Earthworms can survive high-level of exposure by regularly crawling out of the exposure mixture. This practice decreases their contact time with the contaminated soil (Gudbrandsen et al. 2007). Bioaccumulation of Cu, Cd, Pb and Zn by earthworms is well documented and is thought to be in the chloragogenous tissue surrounding the posterior alimentary canal (Li et al. 2010). There are two pathways for the intracellular binding of metals in the chloragogenous tissue. The one pathway binds metals to insoluble, O-donating, phosphate-rich granules (chloragosomes) while the other pathway binds metals to low molecular, S-donating ligands (Sizmur and Hodson 2009). Uptake of Hg by earthworms could be by dermal route or uptake by the gut; the dermal route was however found to be more important, with more than 96 % of Hg found to be taken up by the dermal route (Hobbelen et al. 2006). Contamination in pore water is more available to earthworms for dermal uptake and thus uptake of metals is considered to be mainly via the dermal route. A different fraction of the heavy metal contaminants was present in the gut due to dietary intake because ingested materials are buffered to near neutral pH (Kamitani and Kaneko 2007). These studies typically report on the accumulation of heavy metals from contaminated soil but very few have measured uptake from water (Ernst et al. 2008; Sizmur and Hodson 2009; Calisi et al. 2013; Windmöller et al. 2015). As dermal uptake is very important, determining the accumulation of the metal fraction by water could provide strong evidence of metal mobility and availability (Sizmur and Hodson 2009). The bioavailability of pollutants in soil can be influenced by factors such as pH, cation exchange capacity as well as organic content. In previous reports where earthworms were placed in contaminant Cu bearing solutions, the focus was on differentiating between uptake via pore water and the dermal route (Arnold et al.^2003^; Steenbergen et al. 2005).

In the present study uptake of Hg by earthworms was quantitatively evaluated by placing earthworms in an aqueous/soil controlled microcosm as well as in a dry soil microcosm to assess uptake and bioaccumulation via the important dermal route as well as the gut route. The impact of dissolved HA on the bioavailability of Hg^2+^ was investigated under varying physical conditions of temperature and pH in controlled laboratory experiments.

## Experimental

### Test organism, study soil, aqueous phase and sample preparation

**Fig. 1:**
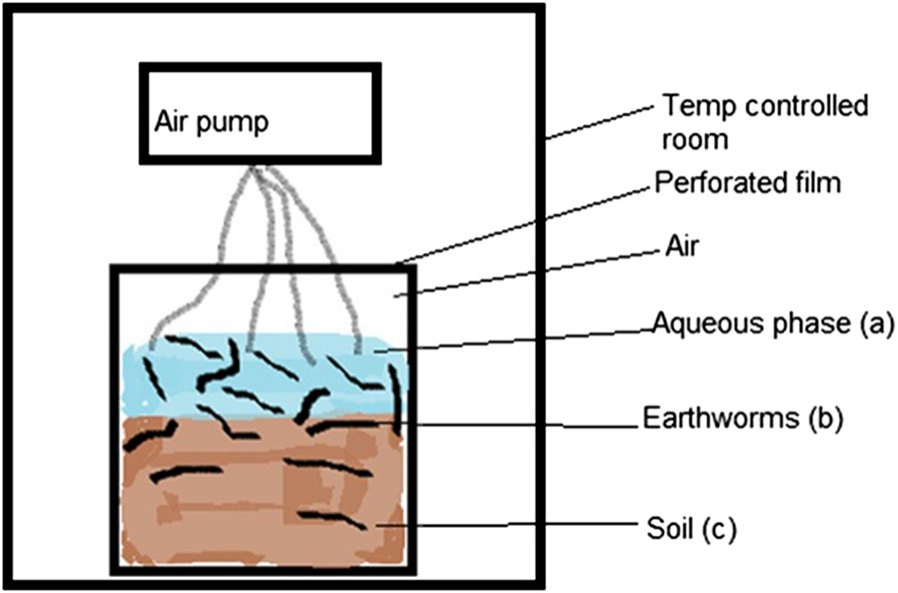
Schematic diagram of the experiment to determine the uptake of Hg by earthworms in the presence of HA

The experiment microcosm (2 L beaker) containing the aqueous phase, soil and earthworms were placed in a temperature controlled room and the experimental microcosms were exposed to a photo-period of 16 h of light (Fig. 1). The experimental set-up containing the aqueous phase required that air be bubbled through, for the preservation of the earthworms. The microcosms were also continually aerated after addition of the earthworms. The earthworms were not fed and the water was not changed in the microcosms over the 5 day duration of the experiments to avoid any factors associated with changes in soil geochemistry. Triplicate samples of all experiments were prepared and analysed separately by direct mercury analyser (DMA).

The aqueous phase (Fig. 1a) was prepared by taking a saline solution, prepared from distilled water and NaCl to a final concentration of 35 µg/mL, spiking it with Hg^2+^ to 5 µg/mL. The pH of this solution was adjusted using acetic acid/ammonium buffer, 5.5, 7 and 8 respectively, in separate experiments as reported in Tables 2, 3 and 4. Soil pH, per se, was not measured as an experimental variable. After the aqueous phase was prepared, it was added on top of the soil layer and allowed to filter through and equilibrate, before the earthworms were introduced to the system.

Earthworms *Eisenia andrei* was the biota selected for the study of the uptake of Hg^2+^ in the presence of HA under different physical conditions. The earthworms were at least 6 weeks old adults of uniform size with well-developed clitella. They were allowed to depurate (the earthworms were left on damp filter paper in petri dishes without food to empty their guts) for 1 day before adding them to the experimental microcosms (30 earthworms/microcosm). During the experiment the earthworms were not fed, and in this way absorption via dermal and gut route was controlled to reflect only what was introduced to the microcosm for experimental purposes (Fig. 1b). After sampling the earthworms were not depurated as the experiment was designed to look at the distribution across all phases and gut content was part of the experimental data.

The soil layer (Fig. 1c) for each experiment was prepared by placing 6-cm layer (600 g) of previously cleaned soil in 2 L beakers. The HA solution, prepared from a soluble form of HA (Sigma Aldrich Catalogue number H1, 675-2 Lot.:S33786-057) was added to a final calculated concentration of 5 µg/g HA in the microcosm and left for 12 h to equilibrate at 4 °C, after which the aqueous phase and earthworms were added (wet microcosm). A control sample was prepared in the same way but without the addition of HA solution.

The sampling period of 5 days was based on a literature precedent from a similar investigation. Laboratory experiments carried out with amphipods, (Lawrence and Mason 2001) showed that a 6-day time period did not significantly affect invertebrate survival (>90 % average survival). The assumption was that treatment used in this study would not affect the percentage survival and growth of earthworms.

**Fig. 2:**
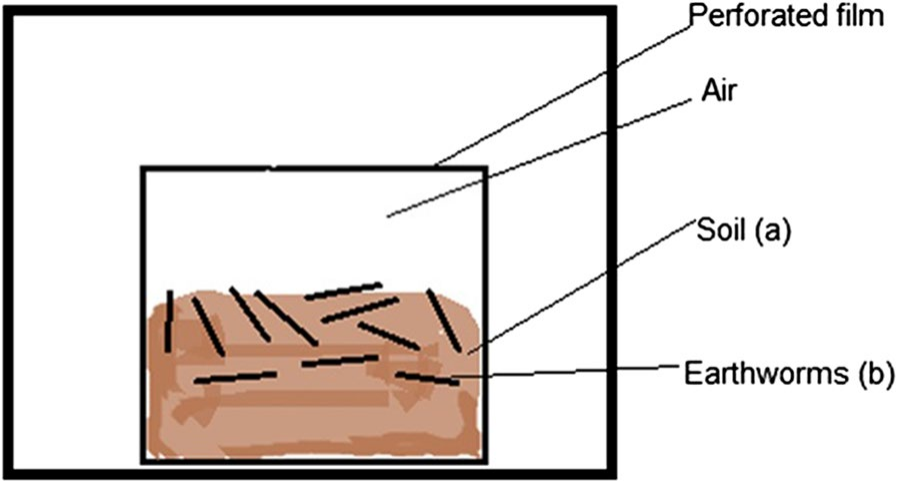
Schematic diagram of the experiment to determine the uptake of Hg by earthworms in the presence of HA

In a separate experiment (dry microcosm) 6-cm layer (600 g) of previously cleaned soil was placed in 2 L beakers (Fig. 2a). The soil was spiked with 0.5 µg/g Hg^2+^ as before and the same concentration HA was used in the 12 h equilibration at 4 °C, before the earthworms were added (Fig. 2b). The control sample for the dry microcosm was prepared in the same way but without adding the HA. Triplicate samples of all experiments was prepared and analysed separately by direct mercury analyser (DMA).

### Analytical procedures for determining the bioaccumulation Hg^2+^ in earthworms

The respective volume of water, soil and earthworm were collected and immediately frozen. All earthworm samples consisted of three earthworms randomly collected at each time interval. Triplicate samples of all phases (aqueous, soil and earthworms) were taken and analysed separately. The sampling period was 5 days; but most samples were taken and analysed within the first 2 days. First experiments in aqueous phase only which were recorded over a nine-day period indicated that most of the complexation took place within the first 48 h. Samples were stored in acid-cleaned Teflon storage containers and refrigerated until subsequent analysis. Analysis was performed using DMA for all samples, to obtain total Hg concentrations and to determine losses of Hg^2+^ from the experimental system.

The frozen samples were transferred to the analytical laboratory and analysed with DMA by the CSIR (Council for Scientific and Industrial Research; Natural Resources and Environment) as a paid service. It is the standard method employed by the CSIR for total mercury analysis. No pre-treatment of the sample was required and the method was suitable for solid and liquid samples. Because no digestion of the sample was needed there was no need for corrosive chemicals and the production of hazardous waste was minimal. The method also has a very good detection limit (Ipolyi et al. 2004). A dual cell atomic absorption spectrometer (AA) was used to determine elemental Hg absorption at a wavelength of 253.65 nm. Triplicate measurements were run on all of the samples, and standard reference materials were run between every 8 samples. A 0.2–0.5 g sample was placed into the sample boat, the sample was weighed, dried for a set period and then thermally decomposed. This process heated the sample to release the mercury and the vapours were carried by oxygen flow to an amalgamator for selective trapping of mercury on a gold trap. After a short heating period, the mercury released was measurement by atomic absorption (Ipolyi et al. 2004; Kading et al. 2009). The certified reference material used was Tort-2 and the Hg concentration found (237.37 ± 31.19) was in good agreement with the certified value at a 95 % confidence level.

## Results and discussion

### Determination of the complexation of Hg^2+^ with HA in aqueous phase, soil and earthworms at a fixed temperature of 293.15 K and variable pH

The complexation of Hg by earthworms in the absence of HA was done, at pH 5.5 only. For this control experiment, the Hg^2+^ concentration in aqueous phase was observed to decrease at the same time as the concentration of Hg^2+^ in the soil and the earthworms increased.

**Fig. 3:**
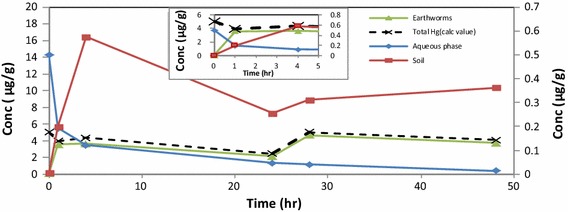
Distribution of Hg^2+^ in aqueous phase, soil and earthworms at pH 5.5 at 293.15 K without any HA present. Earthworm and total Hg values are displayed on primary axis while the aqueous phase and soil values are on the secondary axis

From the time based studies over a period of 48 h, the uptake of mercury followed a time dependent profile during the first 0–5 h. The distribution of mercury between soil, earthworms and aqueous phase in the control experiment, was clearly established by the trends observed from DMA analysis of the appropriate samples for mercury content (Fig. 3).

The concentration of Hg^2+^ in the earthworms increased to a maximum of 74.1 %, within the first 1 h, after which a steady state was reached (Table 1). From the total amount of mercury available in the aqueous phase (5 µg/g), the maximum concentration of Hg^2+^ absorbed by earthworms was at 4 h (3.64 µg/g). The maximum adsorption measured for soil was 0.58 µg/g and the remaining Hg^2+^ in the aqueous was 0.12 µg/g. Hence, applying mass balance principles to the concentration distribution in earthworms at time = 1 h (79.2 %) and at time = 4 h (86.8 %) we can account for the total distribution of Hg^2+^ in the system (Hg_aqueous phase_ + Hg_soil_ + Hg_earthworms_) (dotted line).

**Table 1:** DMA measurements of Hg^2+^ uptake control experiment for soil, earthworms and remaining in aqueous phase

pH values	Time (h)	Concentration (%) remaining in aqueous phase	Concentration (%) in soil	Concentration (%) in earthworms
pH 5.5	1	3.9	4.0	71.4
pH 5.5	4	2.4	11.6	72.8
pH 5.5	24	0.9	5.1	43.1

The complexation experiments were then repeated in the presence of HA, at three different pH values (5.5, 7.0 and 8.0) to observe the effect of pH on mercury uptake.

**Fig. 4:**
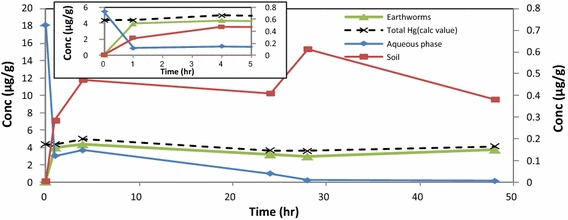
Distribution of Hg^2+^ in aqueous phase, soil and earthworms in the presence of HA at pH 5.5 at 293.15 K. Earthworm and total Hg (calc) values are displayed on primary axis while the aqueous phase and soil values are on the secondary axis

**Fig. 5:**
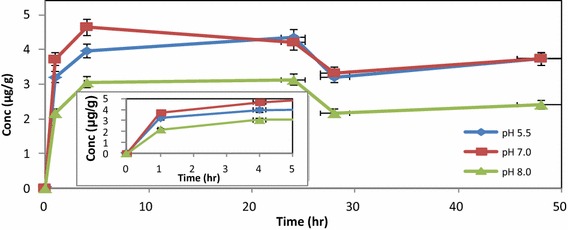
Distribution of Hg^2+^ in earthworms in aqueous phase, at different pH values at 293.15 K (*error bars* represent SE, n = 3)

Following the same data analysis approach as for control experiment, the distribution of mercury between soil, earthworms and aqueous phase in the presence of HA, could account for 94.1 % (after 1 h) and 99.4 % (after 4 h) of the total Hg injected into the biosystem, initially (Fig. 4). Hence, we can account for the total distribution of Hg^2+^ in the system using the mass balance approach, based on measurements of Hg done by DMA (dotted line). The high correlation for mercury distribution is directly attributed to the complexation effect of the HA at pH 5.5 (Fig. 5).

Similar distribution profiles for Hg in the presence of HA were obtained at pH 7.0 and 8.0. The proportion of Hg^2+^ in the different samples, at these pH values were tabulated as the mass balance distribution (Table 2).

**Table 2:** Distribution profile of Hg^2+^ in presence of HA in soil, earthworms and remaining in aqueous phase

pH values	Time (h)	Concentration (%) remaining in aqueous phase	Concentration (%) in soil	Concentration (%) in earthworms
pH 5.5	1	14.2	5.7	74.2
pH 5.5	4	10.9	9.5	79.0
pH 5.5	24	0.8	8.2	87.0
pH 7.0	1	3.5	4.8	74.3
pH 7.0	4	1.9	4.3	92.3
pH 7.0	24	0.64	5.4	84.0
pH 8.0	1	2.7	9.5	43.4
pH 8.0	4	1.1	12.0	78.1
pH 8.0	24	0.5	13.2	62.5

Accumulation of Hg^2+^ in earthworms was less effective at pH 8.0. pH has been identified from literature as being one of the most important parameters that determines uptake of Hg by earthworms (Burton et al. 2006; Ernst et al. 2008). At the beginning of these experiments absorption of Hg was attributed to absorption via the dermal route, due to the skin contact provided by the aqueous phase. However the fluctuation observed in absorption profiles over the extended time period, could be attributed to mixed absorption via dermal as well as the gut route, which are influenced by different physical parameters.

### Determination of the complexation of Hg^2+^ with HA in soil and uptake by earthworms at different temperatures and ambient pH

**Fig. 6:**
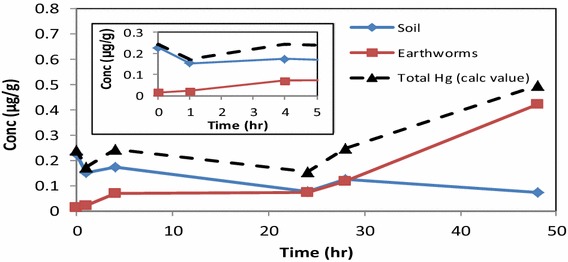
Distribution of Hg^2+^ in soil and earthworms (dry experiment) at 298.15 K in the presence of HA

**Fig. 7:**
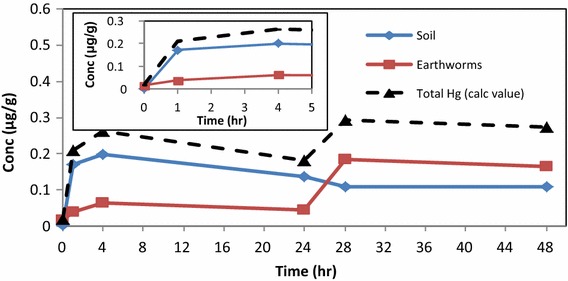
Distribution of Hg^2+^ in soil and earthworms (dry experiment) at 303.15 K in the presence of HA

In subsequent experiments the uptake of mercury by earthworms in dry soil, in the absence of the aqueous phase, was studied at *298.15* *K* and *303.15* *K*. The experimental set-up was the same but the aqueous phase was excluded. The Hg^2+^ concentration in the soil decreased while the concentration of Hg in the earthworms increased rapidly. The data showed an equilibrium condition between 4 and 24 h in the soil, a rapid increase between 24 and 28 h, and then a steady state condition (Figs. 6, 7). The accumulation of Hg^2+^ concentration in the earthworms after 48 h was best observed at 298.15 K, since the higher temperature resulted in expiration of the earthworms.

The distribution of mercury during 0–5 h between soil and earthworms were clearly established. From the total amount of mercury available in the soil (0.5 µg/g), the maximum concentration of Hg^2+^ absorbed by earthworms at 298.15 K after 48 h was (0.42 µg/g) and (0.16 µg/g) at 303.15 K. Accumulation of Hg^2+^ was thus higher at 298.15 K. Hence, applying mass balance principles to the concentration distribution in earthworms at 293.15 and 303.15 K at time = 1 h was (4.4 %) and (7.8 %) respectively and at time = 4 h (14.2 %) and (12.6 %) we can account for the total distribution of Hg^2+^ in the system (Hg_soil_ + Hg_earthworms_) (dotted line). The overall percentages for mercury distribution was again higher when the HA was added.

Accumulation of Hg in earthworms was faster and at higher concentration levels in the aqueous phase. The faster rates represent uptake of Hg^2+^ mainly via the dermal route. Hg distribution profiles based on mass balance of concentrations determined by DMA, account fully for the original value of Hg^2+^ introduced to the bio-system. The best distribution profile (99.4 % accountable) was obtained for aqueous phase experiment at pH 5.5 in the presence of HA. The best distribution profile for soil experiments (i.e. no aqueous phase) was found at lower temperature (84 %). In the soil experiments the absorption takes place mainly via the gut route.

### Kinetics for the complexation of Hg^2+^ with HA in earthworms

**Fig. 8:**
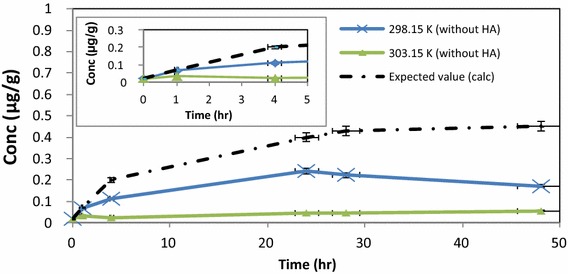
Accumulation of Hg^2+^ in earthworms at 298.15 and 303.15 K without any HA present, in soil experiments (*error bars* represent SE, n = 3)

Hg accumulation trends in earthworms, in the absence of HA (control study) was compared to the expected trend from literature (Veiga et al. 1999; Windmöller et al. 2015). The expected trend is an increase in Hg levels until a maximum after which the concentration remains constant, after which the earthworms expire (Gudbrandsen et al. 2007) (Fig. 8).

**Fig. 9:**
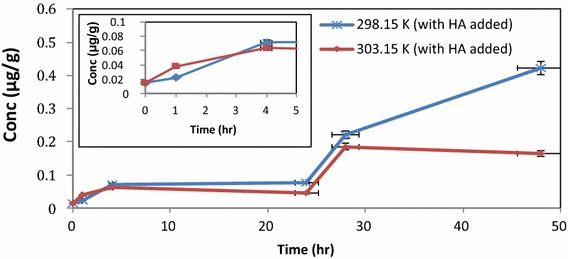
Hg levels in earthworms at 298.15 K (with HA added) and at 303.15 K (with added HA)

The control study confirmed that accumulation of Hg^2+^ in earthworms was favoured by lower temperature and represented uptake of Hg^2+^ mainly via the gut route. Hg absorption experiments, in the presence of HA, showed similar trends at 298.15 and 303.15 K, for accumulation of Hg^2+^ in earthworms up to 30 h after which the lower temperature evaluated, again showed a higher uptake of Hg via the gut route in presence of HA (up to 5 days) (Fig. 9).

In the summary Table 3 the absorption of Hg by earthworms in the aqueous phase experiment (at time 48 h) was highest at 293.15 K at pH 5.5 (lower pH and temperature). For the dry soil experiments Hg absorption was improved at the lower temperature and in the presence of HA (Table 3).

**Table 3:** Summary of Hg^2+^ uptake by earthworms as a function of temperature and pH, at time 48 h

Aqueous phase present				Aqueous phase absent (dry soil experiment)			
293.15 K				298.15 K		303.15 K	
Control (without HA)	pH 5.5 (with HA)	pH 7.0 (with HA)	pH 8.0 (with HA)	Control (without HA)	Sample (with HA)	Control (without HA)	Sample (with HA)
75 %	75 %	75 %	48 %	34 %	84 %	11 %	33 %

The total percentage of Hg remaining in all phases was calculated and divided by the total value of Hg spiked (distribution profile) in an attempt to account for any loss of Hg due to absorption onto container walls or abiotic reduction, i.e. not related to absorption by earthworms and complexation by HA. In this way we have demonstrated that the distribution of Hg in a controlled environment can be fully accounted for. Highest losses were reported at high pH, high temperature and in the absence of HA (Table 4).

**Table 4:** Hg^2+^ percentage distribution profile, based on DMA analysis, at time 48 h

Aqueous phase present				Aqueous phase absent (dry soil experiment)			
293.15 K				298.15 K		303.15 K	
Control (without HA)	pH 5.5 (with HA)	pH 7.0 (with HA)	pH 8.0 (with HA)	Control (without HA)	Sample (with HA)	Control (without HA)	Sample (with HA)
82 %	82 %	80 %	62 %	55 %	99 %	36 %	55 %

### Bioaccumulation factor of Hg in earthworms

Bioaccumulation factors (BAFs) can be used to estimate the bioavailability of metals to earthworm species (Nannoni et al. 2011). The U.S. Environmental Agency (2010) defined the Bioaccumulation factor as ‘The ration of the contaminant in an organism to the concentration in the ambient environment at a steady state, where the organism can take in the contaminant through ingestion with its food as well as through direct content’. It is calculated as the ratio of the Hg concentration in the earthworm (in µg/g dry wt) to the total soil Hg content (in µg/g dry wt) (Álvarez et al. 2004; Rodríquez Álvarez et al. 2014; Nannoni et al. 2011). Previous studies (Burton et al. 2006; Nannoni et al. 2011) found BAF to be <1 which is in agreement with value obtained in the present study (Table 5).

**Table 5:** Bioaccumulation factor of Hg by earthworms at different temperature and pHs after 48 h

Aqueous phase present				Aqueous phase absent (dry soil experiment)			
293.15 K				298.15 K		303.15 K	
Control (without HA)	pH 5.5 (with HA)	pH 7.0 (with HA)	pH 8.0 (with HA)	Control (without HA)	Sample (with HA)	Control (without HA)	Sample (with HA)
0.75	0.75	0.75	0.48	0.34	0.84	0.11	0.33

### Rate constants for the complexation of Hg^2+^ with HA in earthworms, for first order reaction

**Fig. 10:**
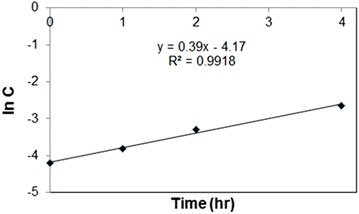
Rate constant of the bioaccumulation of Hg by earthworms with HA present at 298.15 K

Rate constants (k) (bioaccumulation rates) were calculated with first-order kinetic model (Gấrdfeldt 2003; Nannoni et al. 2011). A graph of ln conc vs time (h) was constructed and k evaluated from the slope. A good fit was obtained with r^2^ > 0.99 (Fig. 10).

Values obtained in this study ranged from 0.2 to 1 h^−1^. The highest values obtained were for rate constants calculated from low temperature and pH data for complexation of Hg^2+^ with HA present (Table 6).

**Table 6:** Summary table of rate constant (hr^−1^) calculated for all complexation of Hg with HA

Aqueous phase present				Aqueous phase absent (dry soil experiment)			
293.15 K				298.15 K		303.15 K	
Control (without HA)	pH 5.5 (with HA)	pH 7.0 (with HA)	pH 8.0 (with HA)	Control (without HA)	Sample (with HA)	Control (without HA)	Sample (with HA)
0.4 h^−1^	0.9 h^−1^	1 h^−1^	1 h^−1^	0.4 h^−1^	0.4 h^−1^	0.2 h^−1^	0.3 h^−1^

### Body burden calculations in earthworm

The soil metal content has a huge impact on earthworm body burden. Regression models have been used to calculate the body burden of earthworms from contaminated soils. Body burden calculations relate metal concentrations to soil metal concentrations (Nahmani et al. 2007). Very few studies have been published on determination of the body burden of mercury from soil metal content, hence direct comparison is difficult. However, values for other heavy metals are available e.g. Cd = 0.47 ± 0.12 and Pb = 0.27 ± 0.15 (Nahmani et al. 2007). Predictions of body burden from soil metal content are log-linear regressions of the form:

$${\text{Log}}\,{\text{M}}_{\text{ew}} = a\log \,{\text{M}}_{\text{s}} + b$$

where M_ew_ = concentration of metal in the earthworm (µg/g); M_s_ = concentration of metal in the soil (µg/g); *a* and *b* are constants.

**Fig. 11:**
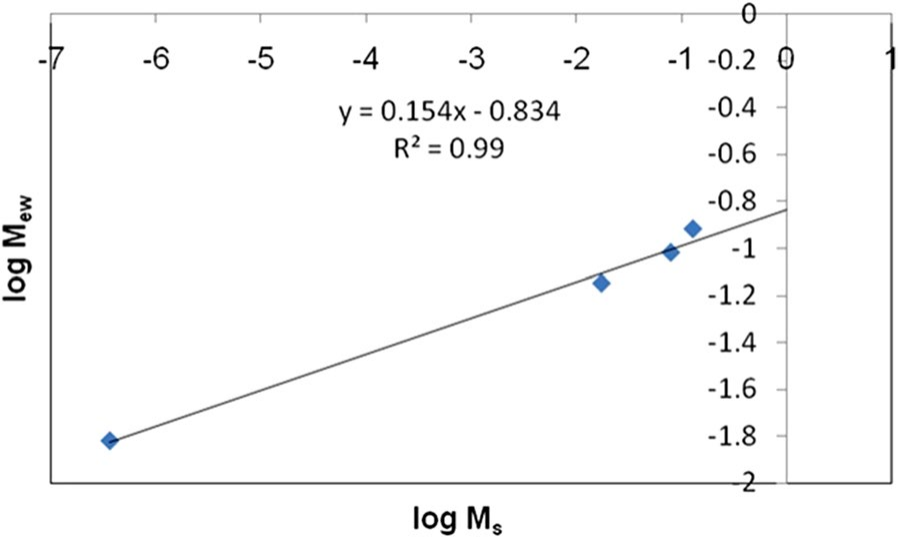
Determination of body burden constants from soil Hg content

Body burdens where calculated by plotting M_ew_ versus M_s_ with *a* = *slope* and *b* = *intercept* (Nahmani et al. 2007). The slope of the regression equation is metal dependent (Corp and Morgan 1991; Heikens et al. 2001) (Fig. 11).

Following the literature precedent we have calculated values for *a* and *b* from the respective linear plot obtained for Hg^2+^. The values for *b* were observed to be relatively constant but greater variation was observed in the value for *a*. Variation in *a* could be due to the absence/presence of HA and the difference in temperature (Table 7).

**Table 7:** Body burden constants for the complexation of Hg with HA in dry experiments with earthworms at 298.15 K and 303.15 K

	298.15 K		303.15	
	Control (without HA)	Sample (with HA)	Control (without HA)	Sample (with HA)
*a*	0.15 ± 0.07	0.10 ± 0.04	0.05 ± 0.02	0.38 ± 0.11
*b*	0.83	0.82	0.85	0.80

The values of *a* for body burden of Hg^2+^ at the lower temperature was comparable to values of *a* determined for other metal species. As noted before, the higher temperature was not tolerated well by the earthworms and resulted in early expiration and hence this factor could influence the body burden data.

## Discussion

Earthworms are important bioindicators of soil contamination. Bioavailability of Hg depends on the speciation, which in turn determines the toxicity, transport, and residual time of the metal in the environment. Abiotic speciation is mainly due to mercury-ligand formation. Previous studies (Burton et al. 2006; Ernst et al. 2008) have found that earthworms could survive highly contaminated soil of up to 22 µg/g Hg and concentrations of up to 6.5 µg/g Hg have been found in earthworms. Colacevich et al. (2011) found that long term exposure of earthworms to Hg-contaminated soil of up to 1287 mg/kg dry wt did not cause earthworm mortality. The ability of earthworms to convert Hg^2+^ to MeHg was also indicated by the speciation results obtained by Santoyo et al. (2011). Veiga et al. (1999) and Kaschak et al. (2014) found that methylation could occur in the gut of earthworms, but upon analysis found only about 1 % MeHg in the total Hg in the tissues.

The detailed kinetic approach that was demonstrated in our work makes a noteworthy contribution to the understanding of the kinetics of mercury uptake by earthworms. Mercury distribution profiles as a function of time. Bioaccumulation factors (BAFs), first order rate constants and body burden constants for mercury uptake under selected conditions of temperature, pH as well as via the dermal and gut route were evaluated in one comprehensive approach. Body burden constants for mercury uptake by earthworms were determined in this work and are reported here for the first time, since no previous reports for reference values were found. This work therefore represents a major contribution to the available knowledge in the evaluation of mercury uptake by earthworms.

## Conclusion

Trends in the uptake of Hg^2+^ by selected invertebrates, earthworms *Eisenia andrei*, were evaluated at different time interval, up to 5 days. Hg analysis of soil, water and earthworm samples was done using standard DMA method. Uptake of Hg by earthworms at 293.15 K was almost immediate, and concentration of up to 75 % of the original spiked value was found at pH 5.5 and pH 7.0 after 48 h. The control sample also showed the same swift uptake and high amount of Hg in the earthworms. The rate constant was determined to be 0.4 in the control sample. In the samples where HA was present in the biosystem, the rate constant was 0.8–1 and Hg accumulation was favoured by low pH and low temperature. The body burden constants determined, were in good agreement with values reported for other divalent metal species.

In the samples containing only the soil and earthworms (no aqueous phase) at 298.15 K the Hg uptake was higher (84 %) in the sample containing the HA than in the control (34 %). The rate of uptake was the same with rate constant of 0.4. The control sample (no HA added), thus showed a similar trend over time, but reduced concentration accumulation of HA. This data suggests that bioavailability of Hg is enhanced through complexation with HA, thereby increasing the uptake of Hg by earthworms. Total Hg unaccounted for was only 1 % at 297.15 K in the presence of HA, but 45 % in control at 48 h.

We have clearly demonstrated that the uptake of Hg was influenced by pH, temperature and HA as supported by calculated values of BAF (0.1–0.8) and rate constants from (0.2 to 1). The body burden of Hg was found to be a = 0.05–0.4 and *b* was found to be constant at 0.8. The higher temperatures and pH were not favourable for uptake of Hg^2+^ in earthworms or indeed survival of the earthworms.
